# Identification and Validation of Six Autophagy-related Long Non-coding RNAs as Prognostic Signature in Colorectal Cancer

**DOI:** 10.7150/ijms.49449

**Published:** 2021-01-01

**Authors:** Lin Cheng, Tong Han, Zheyu Zhang, Pengji Yi, Chunhu Zhang, Sifang Zhang, Weijun Peng

**Affiliations:** 1 Department of Integrated Traditional Chinese & Western Medicine, Second Xiangya Hospital, Central South University, Changsha, Hunan 410011, P.R.China; 2 Department of General Surgery, The Second Xiangya Hospital, Central South University, No.139 Middle Renmin Road, Changsha, Hunan410011, P.R. China.; 3 Department of Integrated Traditional Chinese & Western Medicine, Xiangya Hospital, Central South University, Changsha, Hunan 410008, P.R.China

**Keywords:** Autophagy, colorectal cancer, long non-coding RNA, prognostic signature

## Abstract

Colorectal cancer (CRC) is a commonly occurring tumour with poor prognosis. Autophagy-related long non-coding RNAs (lncRNAs) have received much attention as biomarkers for cancer prognosis and diagnosis. However, few studies have focused on their prognostic predictive value specifically in CRC. This research aimed to construct a robust autophagy-related lncRNA prognostic signature for CRC. Autophagy-related lncRNAs from The Cancer Genome Atlas database were screened using univariate Cox, LASSO, and multivariate Cox regression analyses, and the resulting key lncRNAs were used to establish a prognostic risk score model. Furthermore, quantitative real-time polymerase chain reaction (qRT-PCR) analysis was performed to detect the expression of several lncRNAs in cancer tissues from CRC patients and in normal tissues adjacent to the cancer tissues. A prognostic signature comprising lncRNAs AC125603.2, LINC00909, AC016876.1, MIR210HG, AC009237.14, and LINC01063 was identified in patients with CRC. A graphical nomogram based on the autophagy-related lncRNA signature was developed to predict CRC patients' 1-, 3-, and 5-year survival. Overall survival in patients with low risk scores was significantly better than in those with high risk scores (P < 0.0001); a similar result was obtained in an internal validation sample. The nomogram was shown to be suitable for clinical use and gave correct predictions. The 1- and 3-year values of the area under the receiver operating characteristic curve were 0.797 and 0.771 in the model sample, and 0.656 and 0.642 in the internal validation sample, respectively. The C-index values for the verification samples and training samples were 0.756 (95% CI = 0.668-0.762) and 0.715 (95% CI = 0.683-0.829), respectively. Gene set enrichment analysis showed that the six autophagy-related lncRNAs were greatly enriched in CRC-related signalling pathways, including p53 and VEGF signalling. The qRT-PCR results showed that the expression of lncRNAs in CRC was higher than that in adjacent tissues, consistent with the expression trends of lncRNAs in the CRC data set. In summary, we established a signature of six autophagy-related lncRNAs that could effectively guide clinical prediction of prognosis in patients with CRC. This lncRNA signature has significant clinical implications for improving the prediction of outcomes and, with further prospective validation, could be used to guide tailored therapy for CRC patients.

## Introduction

Colorectal cancer (CRC) is the third most common cancer in the world. More than 1.2 million new cases are diagnosed each year, about 45% of which are fatal^1^, ^2^. Survival rates of CRC patients have increased to some extent because of advances in treatment methods and earlier diagnosis^3^-^5^. In clinical settings, molecular subtype, histological grade, and tumour stage are evaluated to predict prognosis^6^, ^7^. However, these clinicopathological characteristics do not provide sufficiently accurate information, which may result in incorrect prognosis judgements. Patients at low risk might receive excessive or unnecessary treatment, whereas others may progress to metastasis or relapse because of insufficient treatment^8^. Thus, it is important to find new molecular markers for predicting the prognosis of patients with CRC and determining the appropriate treatment.

With recent improvements in high-throughput sequencing technology, combinations of bioinformatics and microarray data have been adopted worldwide for the diagnosis of a variety of cancers and for the development of prognostic biomarkers. Data mining and multivariate and univariate Cox regression analyses have been used to develop gene signatures comprising several related genes. Such gene signatures have been used to enable accurate survival prediction, individualized therapy, and molecular diagnosis, as their prediction accuracy is better than that of a single biomarker^9^, ^10^. In this study, we used a bioinformatics approach to establish an autophagy-related long non-coding RNA (lncRNA) signature for predicting the prognosis of patients with CRC.

Autophagy is an ancient and greatly conserved catabolic process, comprising a series of molecular events that cause the formation of an autophagosome, which engulfs intracellular material and finally fuses with the lysosome to degrade its contents. Autophagy has been shown to be activated robustly in various malignant tumour types and contributes to the development and progression of cancers^11^-^14^. Furthermore, accumulating data have revealed that the autophagy process in cancer cells is regulated by lncRNAs, which are RNA molecules with length greater than 200 bp and no protein-coding function^15^-^17^. For example, Malat1 may activate autophagy and participates in tumorigenesis in a number of cancer cells, and HOTAIR may activate autophagy in hepatocellular carcinoma^18^, ^19^. In CRC, autophagy has also been reported to be activated by Malat1, which in turn inhibits apoptosis and promotes cell proliferation through sponging miR-101. Another lncRNA, UCA1, was found to be associated with cell autophagy, which it may regulate through the AKT/mTOR signalling pathway^20^-^22^. Autophagy-related lncRNAs have also been shown to possess predictive value with respect to glioma patients' prognosis^23^. However, there is no systematic process to identify autophagy-associated lncRNA signatures for predicting the survival of patients with CRC.

Here, we attempted to establish an autophagy-related lncRNA signature for predicting the prognosis of patients with CRC. The results indicate potential clinical applications of autophagy-related lncRNAs in prognostic stratification and could be used to guide targeted treatment of CRC.

## Materials and methods

### Flowchart of the study process

**Figure 1:**
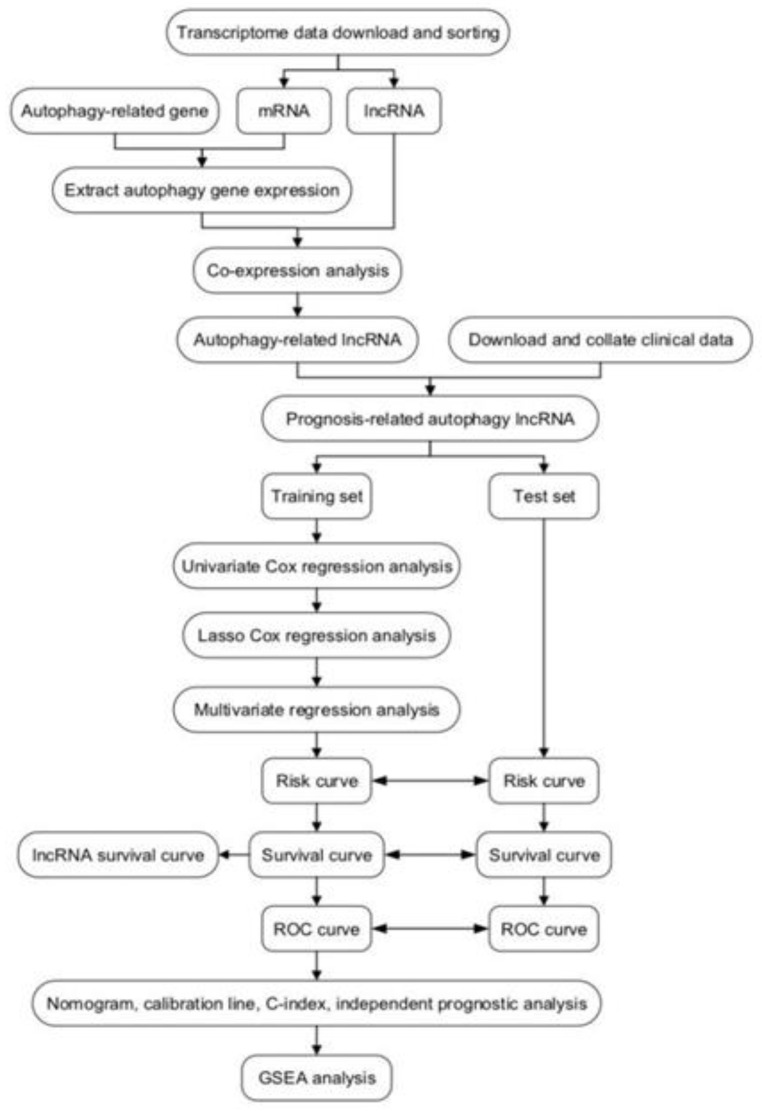
Workflow of identification of survival-related signature in colorectal cancer based on six autophagy-related long non-coding RNAs (lncRNAs). GSEA, gene set enrichment analysis; ROC, receiver operating characteristic.

A detailed workflow is shown in Figure 1. We established and verified a signature of six autophagy-related lncRNAs.

### Data sources and processing

Normalized RNA sequencing data sets were obtained from The Cancer Genome Atlas (TCGA) database (https://cancergenome.nih.gov/), comprising estimated fragments per kilobase of transcript per million mapped reads from 39 non-tumour samples and 398 tumour samples. Clinical data were also derived from TCGA, including age, gender, TNM classification, and pathological staging. Autophagy gene data were downloaded from the Human Autophagy Database (HADb; http://www.autophagy.lu/index.html), and an autophagy-related gene expression matrix was extracted from the mRNA matrix. Then, the “limma” package in the R software was used to for co-expression analysis of the expression matrix for autophagy-related genes and the lncRNA matrix, and autophagy-related lncRNAs were obtained. Pearson correlation analysis was used to calculate correlations between the autophagy-related genes and lncRNAs. A correlation coefficient > 0.3 and P < 0.001 were considered to indicate significance.

### Development and verification of prognostic signature

First, we combined the autophagy-related lncRNA expression matrix with survival data; then, the “survival” R package was used to identify autophagy-related lncRNAs strongly linked to overall survival (OS) via univariate Cox regression analysis; P < 0.01 was considered statistically significant. Subsequently, the TCGA data set was classified randomly into a test set (n = 190) and training set (n = 190). Using the “glmnet” R package, LASSO regression analysis was carried out to remove highly related lncRNAs from the training set, to avoid excessive matching of the signature model. Next, we used the autophagy-related lncRNAs obtained from the LASSO regression analysis to perform multivariate Cox regression analysis. A forward and backward selection algorithm was used to obtain the most appropriate model. Finally, the prognostic signature was constructed by weighting the regression coefficient obtained from the multivariate Cox regression analysis. Risk scores were calculated using the following formula, where x_i_ indicates gene-related expression levels and β_i_ indicates the coefficients associated with gene expression:


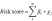


Using this formula, risk scores were calculated for each patient, and the “survminer” R package was used to divide the training group into high- and low-risk groups on the basis of the median risk score. Kaplan-Meier survival curves were plotted with the “survival” R package to evaluate the differences in survival between the high- and low-risk groups. The area under the curve (AUC) of the time-dependent dynamic receiver operating characteristic (ROC) curve and the concordance index (C-index) were used to check the accuracy of the prognostic model. The formula was used in a similar way in the test set to validate its stability.

### Relationship between risk score and clinical characteristics

Clinical characteristics were collected from TCGA, including TNM status, stage, gender, and age, and integrated with the risk score file for each sample derived from TCGA. Subsequently, the “survival” R package was used to carry out multivariate and univariate Cox regression analyses to test whether risk scores were independent of clinical characteristics as prognostic factors. P < 0.05 was regarded as statistically significant. We also used the multi-indicator ROC curve to evaluate the accuracy of the risk score in predicting the survival of patients.

### Construction of a nomogram based on the autophagy-related lncRNA signature

We constructed a nomogram using the “rms” R package, combining the signature of six autophagy-related lncRNAs with clinicopathological risk factors, for use as a quantitative prediction tool to evaluate clinical prognosis. The accuracy of the prediction was tested using a calibration chart to show the difference between predicted survival and actual survival, where the 45° line indicates the best prediction result.

### Gene set enrichment analysis (GSEA)

To determine which pathways were active within the high- and low-risk groups, respectively, GSEA was performed using annotated gene set c2.cp.kegg.v7.0, with symbols.gmt as the reference data set. Gene sets with false discovery rate less than 0.05 after 1000 permutations were considered to be significantly enriched.

### Clinical CRC sample collection

Fifteen CRC samples and paired adjacent samples were taken from patients diagnosed with CRC and undergoing surgery at the Second Xiangya Hospital of Central South University. All specimens were immediately frozen in liquid nitrogen after removal and stored at -80 ℃ for later use. All patients signed informed consent before surgery. This study was approved by the Ethics Committee of the Second Xiangya Hospital of Central South University.

### Quantitative real-time polymerase chain reaction (qRT-PCR) validation

Total RNA was extracted from tissue samples using TRIzol reagent (Invitrogen, Grand Island, NY, USA). RNA quantity and quality were assessed with a NanoDrop ND-1000 spectrophotometer (Thermo Scientific, Waltham, MA, USA), and RNA integrity was evaluated by standard denaturing agarose gel electrophoresis. The RNA was then reverse-transcribed into cDNA using SuperScript III Reverse Transcriptase (Invitrogen) according to the manufacturer's instructions. An Applied Biosystems ViiA 7 Real-Time PCR System and 2× PCR Master Mix (Arraystar) were used for qRT-PCR following the manufacturer's instructions. Relative lncRNA expression levels were calculated using the 2^-ΔΔCt^ method. The primers used and their sequences are listed in Table 1. Data represent the mean of three experiments.

## Results

### Construction of the prognostic model

**Figure 2:**
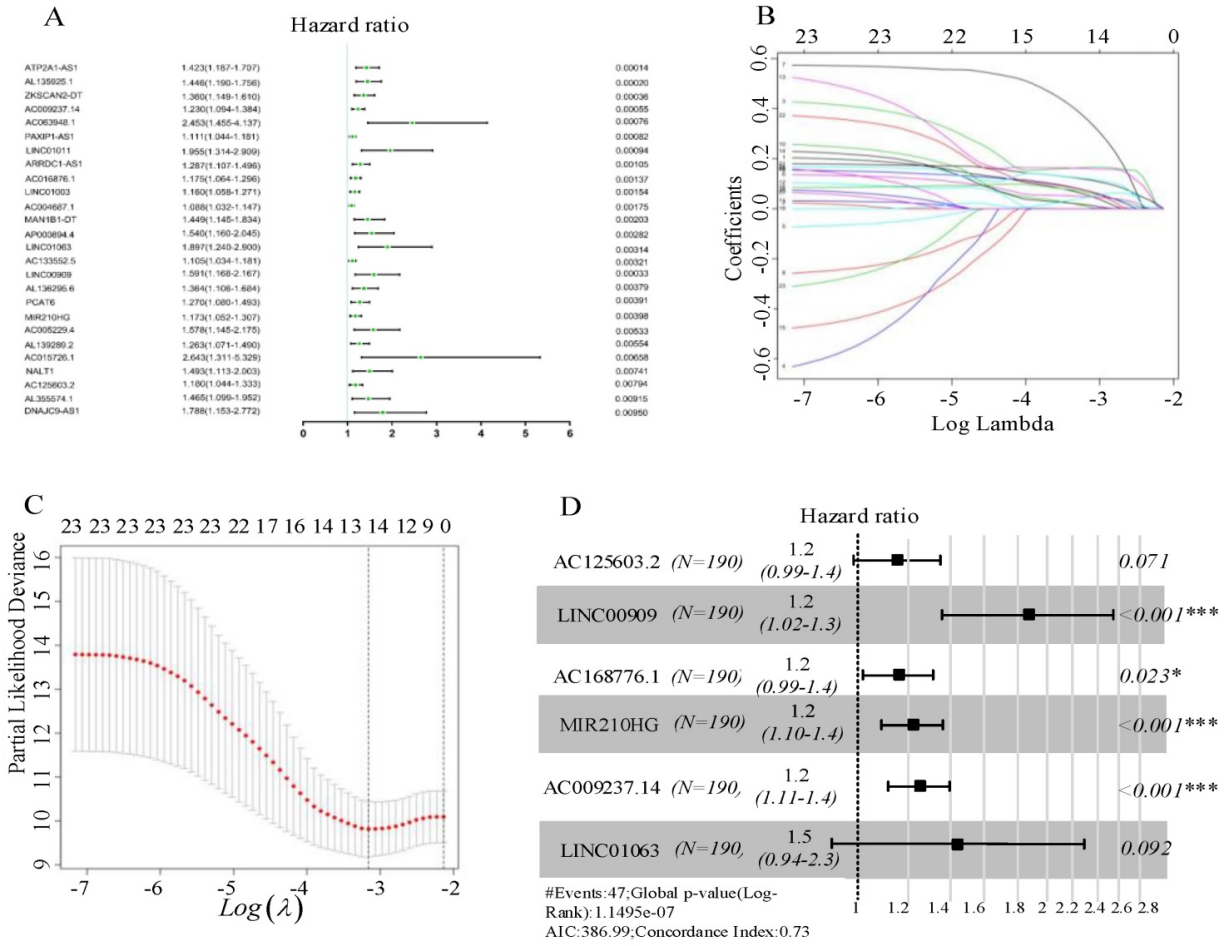
(A) Forest plots of hazard ratios of survival-associated autophagy-related long non-coding RNAs in colorectal cancer. (B) Ten-fold cross-validation for tuning parameter selection in the LASSO model. (C) LASSO coefficient profiles for the 13 prognostic autophagy-related long non-coding RNAs. A vertical line is drawn at the value chosen by 10-fold cross-validation. (D) Six key autophagy-related long non-coding RNAs associated with overall survival in colorectal cancer.

In total, 1247 autophagy-related lncRNAs were identified from TCGA. After combining the expression of the 1247 autophagy-related lncRNAs with survival data for CRC, univariate Cox regression analysis was carried out to identify autophagy-related lncRNAs related to prognosis, resulting in 26 autophagy-related lncRNAs (Figure 2A). Subsequently, we randomly divided the TCGA data set into a test set (n = 190) and training set (n = 190). Using the candidate lncRNAs screened by univariate Cox regression analysis, LASSO regression analysis was performed in the training set, and 13 prognostic autophagy-related lncRNAs for CRC were identified (Figure 2B, C). These lncRNAs were included in the subsequent multivariate Cox regression analysis, which identified six autophagy-related lncRNAs (AC125603.2, LINC00909, AC016876.1, MIR210HG, AC009237.14, and LINC01063). These lncRNAs were considered to have prognostic value and were used to build the predictive model. Hazard ratio (HR) values and 95% confidence intervals (CIs) for the key lncRNAs are presented in Figure 2D. On the basis of the multivariate Cox regression analysis, individual risk scores were calculated using the coefficient-weighted expression levels of the six lncRNAs after the extraction of the coefficient value, according to the following formula:


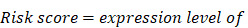
AC125603.2*0.14627597+

LINC00909*0.14627597+AC016876.1*0.62895301+MIR210HG

*0.2088022+AC009237.14*0.22118415+LINC01063*0.38106118

**Figure 3:**
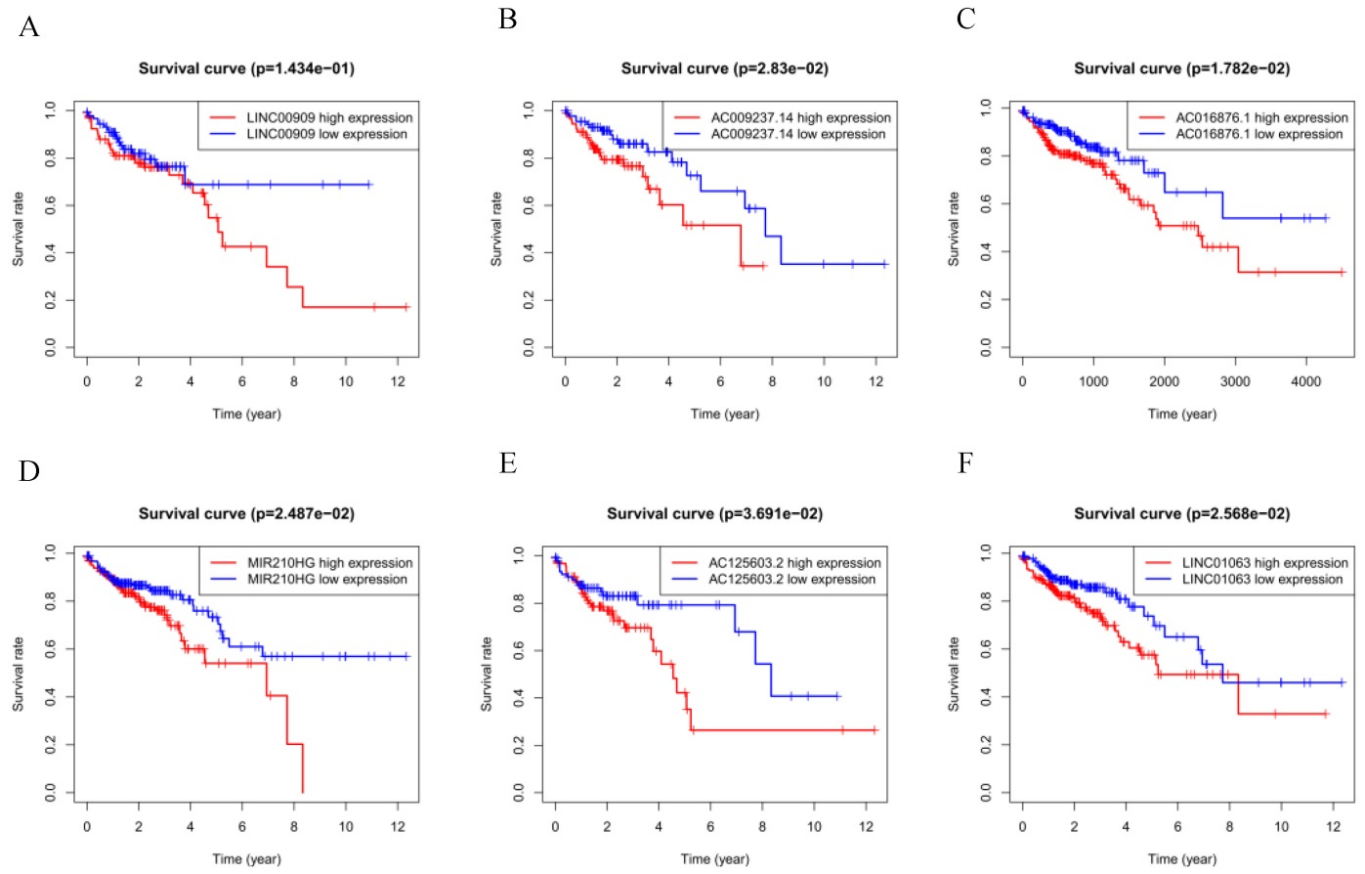
(A-F) Kaplan-Meier survival curves for the six prognostic long non-coding RNAs for colorectal cancer in The Cancer Genome Atlas.

The six autophagy-related lncRNAs were considered to be risk-related lncRNAs and were positively correlated with poor prognosis. The relevant survival curves are shown in Figure 3A-F.

### Verification of the prognostic model

**Figure 4:**
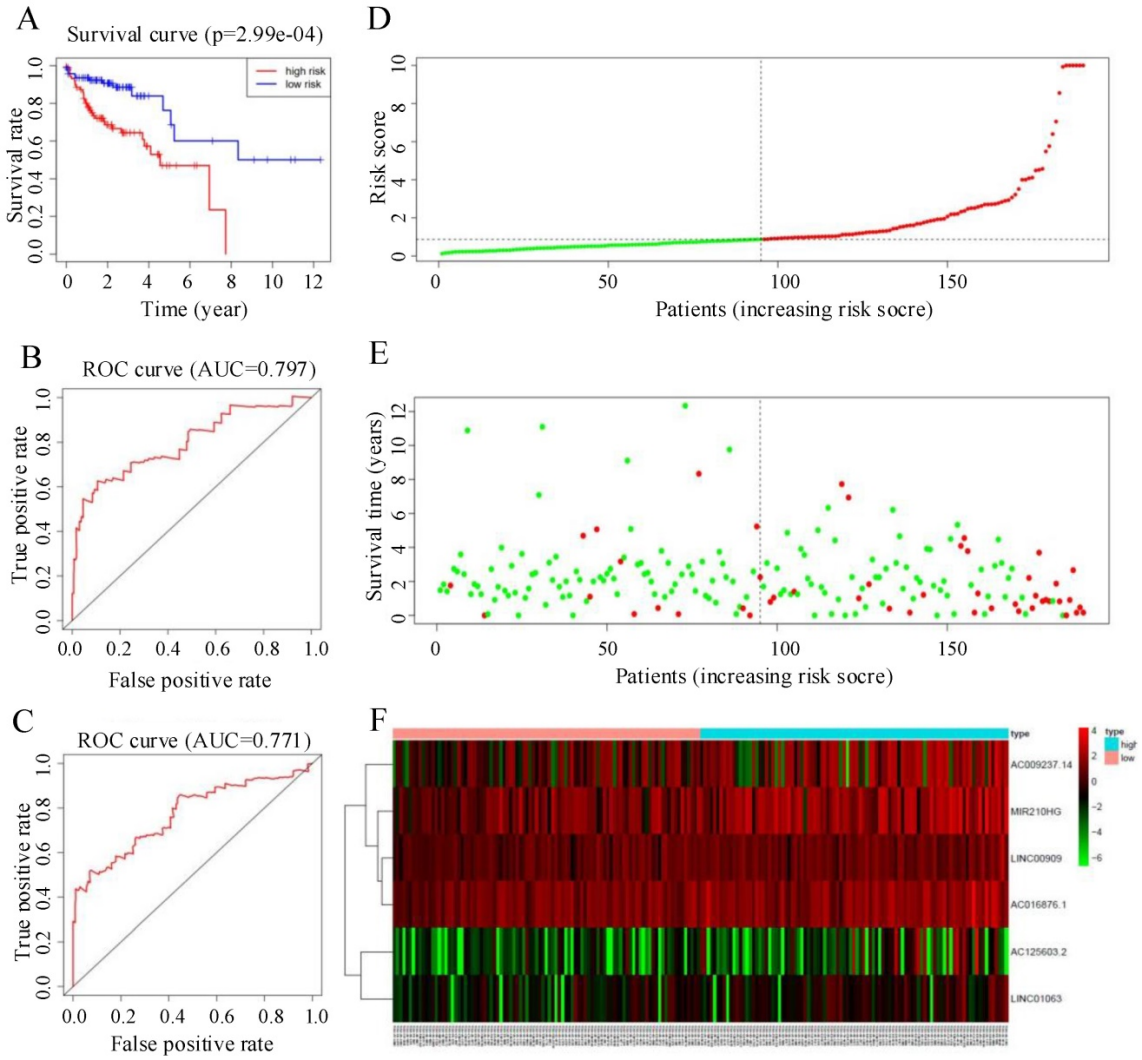
(A) Kaplan-Meier curves for the training group. (B, C) Time-dependent receiver operating characteristic (ROC) curves for overall survival (OS) prediction at 1 year (B) and 3 years (C) in the training group. Time-dependent ROC curves for OS prediction at 3 years. (D) Distribution of patients with high risk score (red) and low risk score (green) in the training set. (E) Survival status of colorectal cancer patients (red dots indicate fatalities, green dots indicate survival) in the training set. (F) Heatmap of six key prognostic lncRNAs in the training set.

The median risk score was 0.88. Individuals whose risk scores were less than 0.88 were assigned to the low-risk group, and those whose risk scores were higher than 0.88 were assigned to the high-risk group. Survival analysis showed that the survival rate of the low-risk group was significantly higher than that of the high-risk group (HR = 1.6, 95% CI = 32.5-67.9, P < 0.05; Figure 4A). ROC curve analysis (Figure 4B, C) gave acceptable AUC values for 1-year and 3-year survival of 0.797 and 0.771, respectively. Figure 4D-F shows the distribution of risk score, survival status of CRC patients, and a heatmap of the six key prognostic lncRNAs.

**Figure 5:**
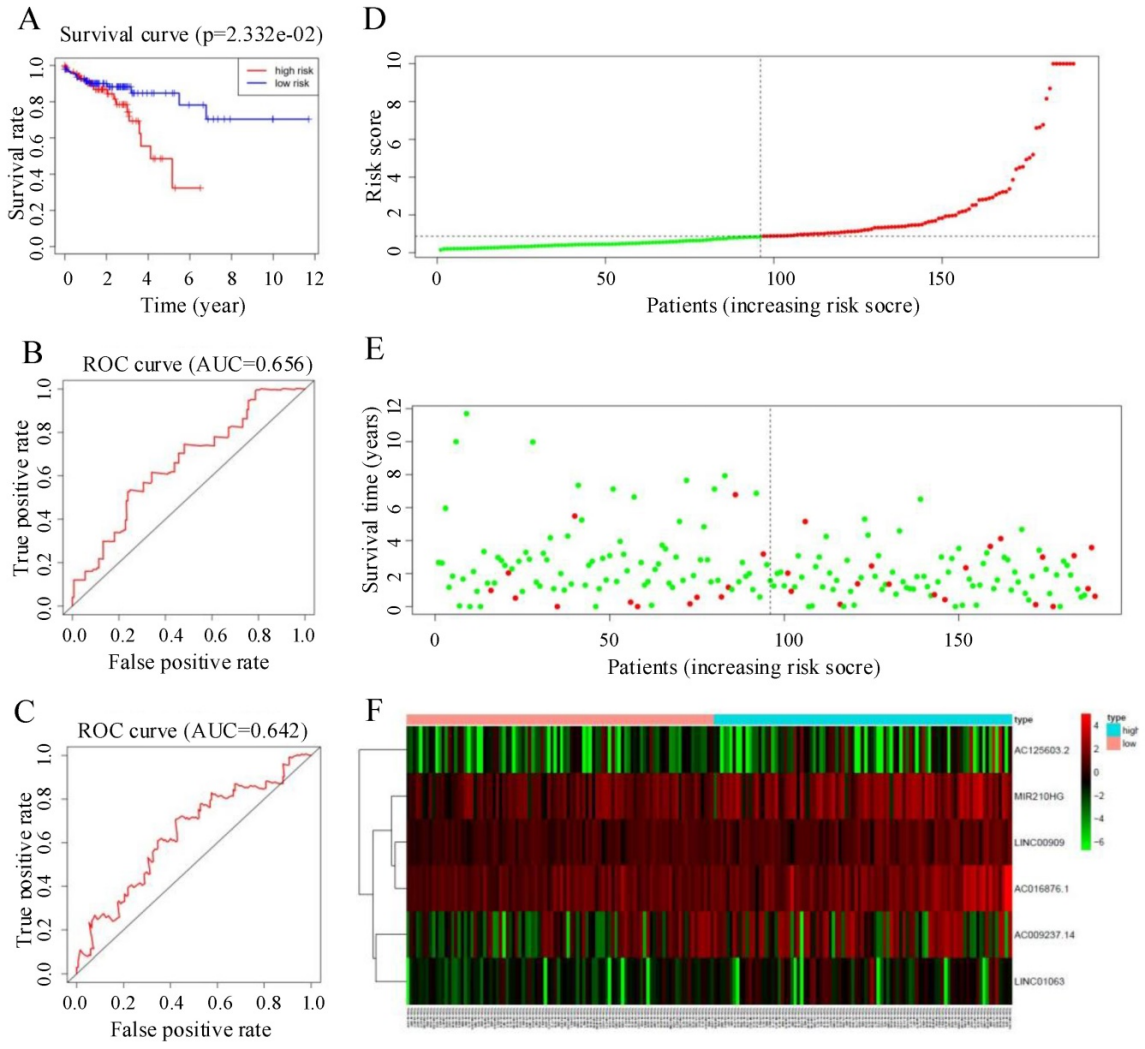
(A) Kaplan-Meier curves for the test group. (B, C) Time-dependent receiver operating characteristic (ROC) curves for overall survival prediction at 1 year (B) and 3 years (C) in the test group. (D) Distribution of patients with high risk score (red) and low risk score (green) in the training set. (E) Survival status of colorectal cancer patients (red dots represent fatalities, green dots represent survival) in the training set. (F) Heatmap of six key prognostic lncRNAs in the test set.

**Figure 6:**
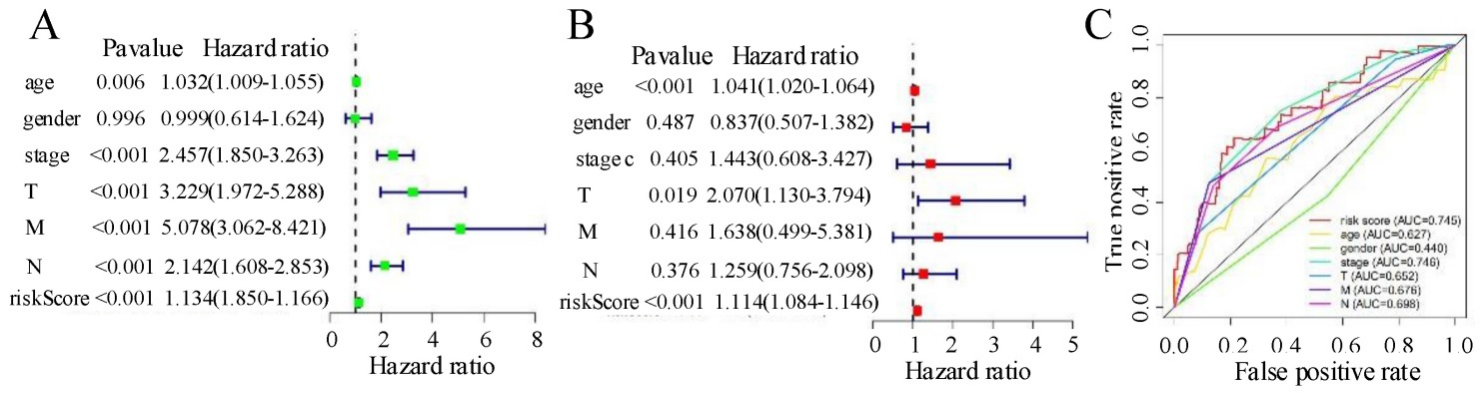
(A) Univariate Cox analyses for risk score, age, gender, tumour stage, and TNM. (B) Multivariate Cox analyses for risk score, age, gender, tumour stage, and TNM. (C) Receiver operating characteristic analysis for 1-year overall survival using the risk score and classical clinicopathologic parameters in The Cancer Genome Atlas cohort. AUC, area under the curve.

To verify the predictive ability of the autophagy-related lncRNA signature, we used the risk score formula to calculate individual risk scores and divided patients in the test set into low-risk and high-risk groups accordingly. Figure 6 summarizes the verification results for the test set. The Kaplan-Meier survival curve showed a significant separation in the test set. The prognosis of the patients in the high-risk group was significantly worse than that in the low-risk group, consistent with the results in the training set (P < 0.05, 95% CI = 30.5-77.2, HR = 1.6, Figure 5A). ROC curve analysis gave acceptable AUC values of 0.656 and 0.642 for 1-year and 3-year survival, respectively (Figure 5B, C). Figure 5D-F shows the distribution of risk score, survival status of CRC patients, and a heatmap of the six key prognostic lncRNAs in the test set; these showed similar trends to those observed in the training set. In summary, the autophagy-related lncRNA signature has the ability to predict OS in CRC.

Using the R software, the C-index was calculated based on the AUC of the ROC curve and used to estimate the probability that the predicted results were consistent with the actual observation results, that is, the accuracy of the model. The C-index values for the verification samples and training samples were 0.756 (95% CI = 0.668-0.762) and 0.715 (95% CI = 0.683-0.829), respectively. Therefore, the risk score model had high accuracy.

### The signature of six-autophagy-related lncRNAs is an independent prognostic factor in CRC

In the univariate Cox analysis, grade, TNM stage, age, and risk score were strongly correlated with OS (Figure 6A), whereas in the multivariate Cox regression analysis, only T stage, age, and risk score were strongly linked to OS (Figure 6B). The prognostic markers based on the six autophagy-associated lncRNAs were independent of other clinical characteristics (e.g., age, gender, TNM stage) as prognostic factors. The multi-index ROC curves showed a 3-year AUC of 0.745 for the risk score, substantially higher than those for N stage (AUC = 0.698), M stage (AUC = 0.676), T stage (AUC = 0.652), and age (AUC = 0.627). These results indicate that the six-autophagy-related lncRNA signature showed acceptable accuracy in predicting patient survival and performed better than other clinical characteristics (including TNM status, stage, and age). The results are shown in Figure 6C.

### Nomogram based on signature of six autophagy-related-lncRNAs for prognostic prediction in CRC patients

**Figure 7:**
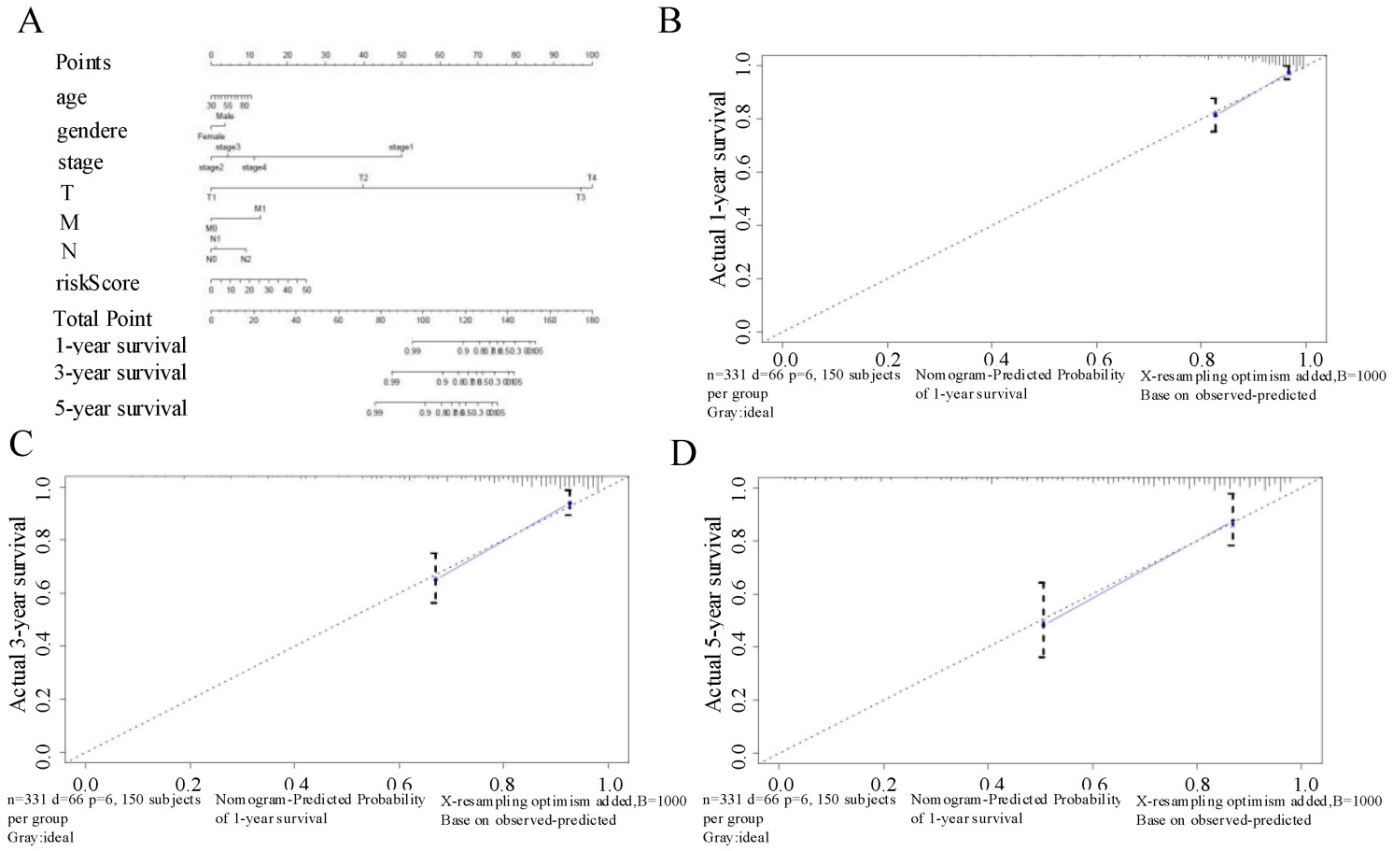
(A) Nomogram based on the signature and clinical information. (B-D) Calibration plots of the predictive accuracy of the nomogram for (B) 1-year survival, (C) 3-year survival, and (D) 5-year survival.

We developed a nomogram using the six autophagy-related lncRNAs and the clinical factors for CRC described above to predict OS at 1, 3, and 5 years (Figure 7A). The calibration chart (Figure 7B-D) showed the best prediction accuracy, and the predicted survival rates were approximately equal to the actual survival rates.

### Gene set enrichment analysis

**Figure 8:**
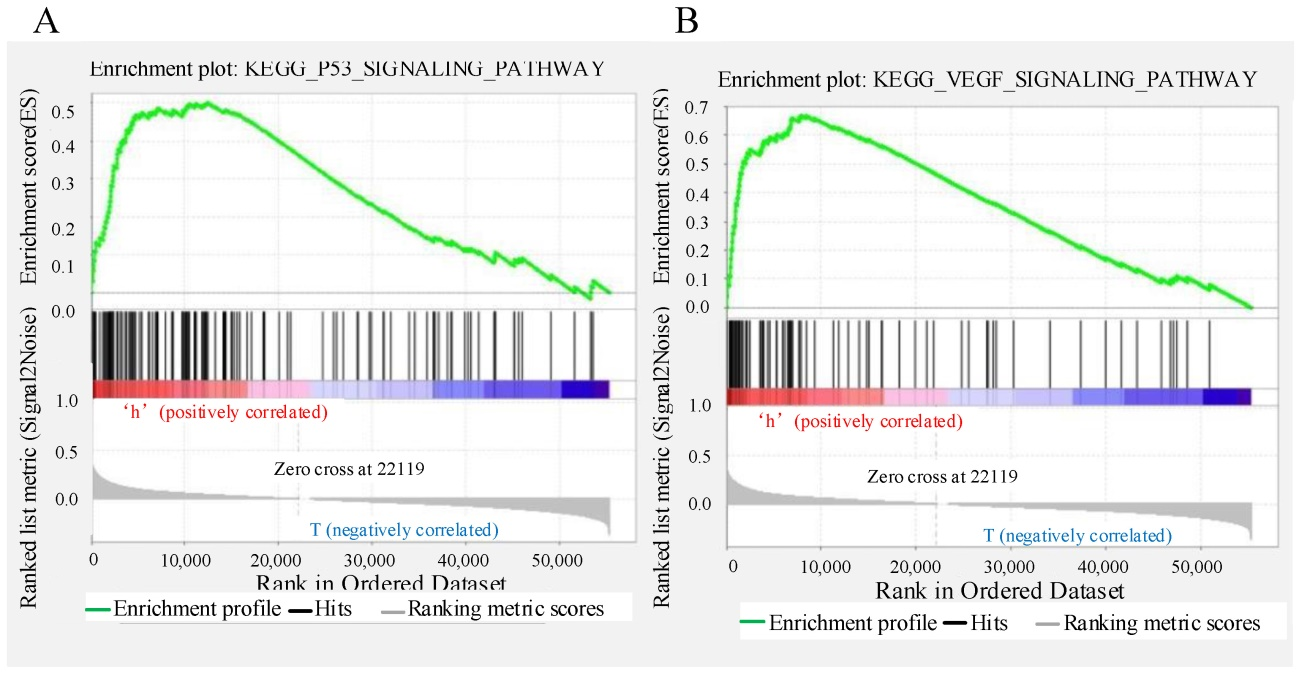
Gene set enrichment analysis indicating significant autophagy-related enrichment based on The Cancer Genome Atlas data set.

The predictive performance of the signature based on six autophagy-related lncRNAs was expected to be linked to the biological functions of the lncRNAs in CRC. To explore the potential mechanism, GSEA was applied to identify the Kyoto Encyclopedia of Genes and Genomes (KEGG) pathways enriched in the low- and high-risk groups. The results showed that “p53 signalling pathway” and “VEGF signalling pathway” were enriched in the high-risk group (Figure 8A-B); these pathways are strongly related to autophagy and the development of CRC.

### Validation of the expression of lncRNAs in CRC samples

**Figure 9:**
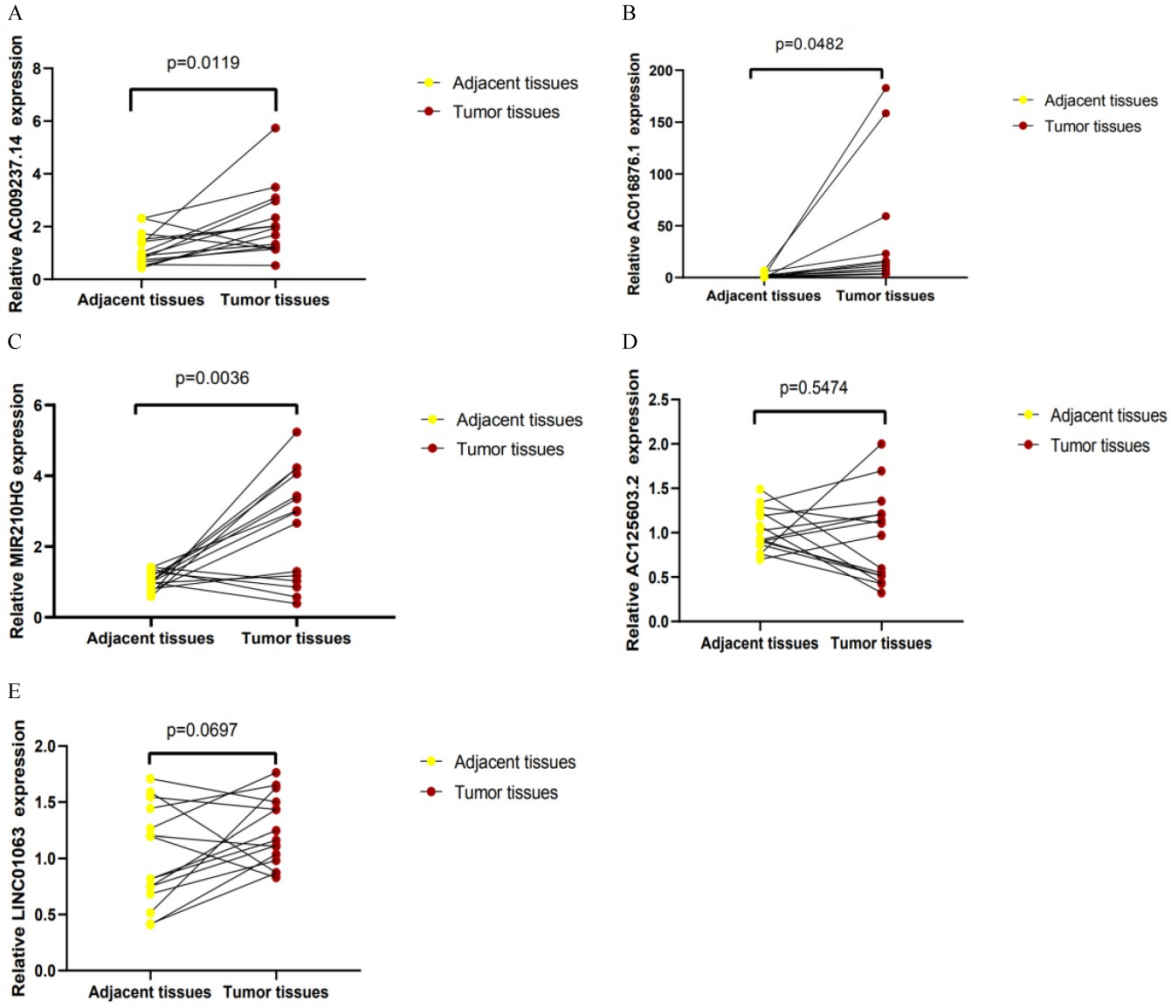
Quantitative real-time polymerase chain reaction results for five lncRNAs: (A) AC009237.14, (B) AC016876.1, (C) MIR210HG, (D) AC125603.2, (E) LINC01063

qRT-PCR was used to detect the expression levels of AC125603.2, LINC00909, AC016876.1, MIR210HG, AC009237.14, and LINC01063 in 15 CRC samples and paired adjacent samples. According to the results, AC016876.1, MIR210HG, and AC009237.14 were significantly upregulated in CRC tissues compared with adjacent tissues, consistent with their expression trends in the TCGA data set (Figure 9A-C) (P < 0. 05). The sequence of LINC00909 is very similar to that of XM-006722500.4 (ZNF407), so it was difficult to distinguish it with the primers we designed. In the tumour tissues, the expression levels of AC125603.2 and LINC01063 were higher compared with those in the adjacent tissues, but this difference did not reach statistical significance (Figure 9D-E) (P > 0. 05).

## Discussion

We obtained autophagy-related genes from TCGA and HADb and used univariate Cox analysis, multivariate Cox analysis, and LASSO regression analysis to construct a signature based on six autophagy-related lncRNAs (AC125603.2, LINC00909, AC016876.1, MIR210HG, AC009237.14, and LINC01063) for predicting OS of patients with CRC. The signature was evaluated in the training set. The survival rate of patients in the high-risk group was substantially lower than that of those in the low-risk group. ROC curve analysis, C-index analysis, and internal verification showed that the risk score system could accurately predict OS in CRC patients. In addition, multivariate Cox analysis showed that the prognostic signature based on autophagy-related lncRNAs was an independent factor after adjusting for clinical features (TNM status, stage, gender, and age). Furthermore, we established a nomogram for predicting the prognosis of CRC patients. The calibration plot showed that the predicted survival was very close to the actual survival, indicating that our nomogram has good predictive performance. Finally, the expression of the lncRNAs included in the signature was validated in clinical samples by qRT-PCR analysis. The results suggested that these six autophagy-related lncRNAs were strongly associated with prognosis in CRC and could be a powerful indicator of clinical outcome in patients with CRC.

The effective prognostic prediction of the six autophagy-related lncRNAs could be related to the biological functions of the lncRNAs in CRC. However, the biological functions of the lncRNAs (AC125603.2, LINC00909, AC016876.1, MIR210HG, AC009237.14, and LINC01063) in our signature had not been reported previously. Therefore, in order to determine the underlying mechanism, we performed GSEA. The results indicated that “p53 signalling pathway” and “VEGF signalling pathway” were enriched in the high-risk group. p53 is one of the most important tumour suppressor genes and is often found to be absent or inactivated in CRC^24^, ^25^. The p53 signalling pathway plays a very important part in regulation of cell growth, proliferation, and apoptosis^26^. When DNA damage to cells is caused by external stimulation or p53-negative tumour cells are reactivated, p53-induced autophagy will occur^27^-^30^. As a transcription factor, p53 in the nucleus can upregulate autophagy by activating the DRAM regulatory factor upstream of mTOR. DRAM1 was the first protein found to be directly related to p53 and autophagy and has an important role in p53-mediated autophagy. Studies have shown that p53 can directly activate the transcriptional expression of DRAM^31^-^33^. Formation of new blood vessels is an important process in tumour growth, metastasis, and transmission. The VEGF pathway is the key regulator of this process and can promote the growth, metastasis, and survival of vascular endothelial cells, resulting in tumour proliferation and malignant prognosis^34^-^36^. Ruan et al. found that during tumour growth, ischemia and hypoxia promote VEGF to activate PI3K, and then activate the PI3K/AKT/mTOR signal transduction pathway^37^-^39^. A conserved serine/threonine protein kinase, mTOR forms a junction of upstream pathways that regulate cell growth, proliferation, and autophagy^40^-^42^. The mechanism by which mTOR regulates autophagy includes the following processes: (1) mTOR kinase directly acts on the Atg protein to regulate the formation of autophagosomes; (2) mTOR-mediated signal transduction acts on downstream effectors, including 4e-bp1 and S6K kinase, to initiate transcription and translation of related genes and regulate autophagy^43^, ^44^. In summary, the six autophagy-related lncRNAs might regulate autophagy through the above pathway, resulting in differences in survival outcomes between groups that were defined using prognostic characteristics.

The current research had several limitations. First, the expression levels of AC125603.2 and LINC01063 in cancer tissues were higher than those in para-carcinoma tissues, but this difference did not reach statistical significance (P > 0.05). This was probably due to the modest effect and small sample size; significant differences may be found with an enlarged sample size. Second, the risk score model needs to be further validated in clinical trials to verify its clinical utility. Besides, the functions and mechanisms of the six autophagy-related lncRNAs need to be further analysed.

In conclusion, we constructed a six autophagy-related lncRNAs signature correlated with CRC prognosis. This model can predict overall survival of patients with CRC after enterectomy and might be useful for the development of individualized treatment for CRC patients, but the feasibility about its use in population needs to be further validation.

## Figures and Tables

**Table 1:** Primers designed for qRT-PCR validation of candidate lncRNAs

Name	Bidirectional primer sequence	Tm (°C)	Product length (bp)
β-actin (H)	F:5' GTGGCCGAGGACTTTGATTG 3' R:5' CCTGTAACAACGCATCTCATATT 3'	60	73
AC009237.14	F:5' GGTCTGTGATTCTGCTGATGG 3' R:5' CCCCTGGAGTCTTTCTTTGA 3'	60	194
AC125603.2	F:5' GCTAAGAGGCTGACGGGTAA 3' R:5' AGCTGGATAATGAATTTGCACT 3'	60	131
AC016876.1	F:5' GCATTTCTCAGCTGCTTCCG 3' R:5' GACGGGGTTTTCCTTGTCCT 3'	60	111
LINC01063	F:5' CCTGAGCCTGGAAGGTGATT 3' R:5' TGACTGAGGTTCGCTGTGAC 3'	60	165
MIR210HG	F:5' GGTTCTGGCTTGCTGACAC 3' R:5' CAACTCGGCTTGGTTATTTC 3'	60	103
